# Editorial

**Published:** 1846-08

**Authors:** 


### THE MEDICAL EXAMINER.

PHILADELPHIA, AUGUST, 1846.

### NATIONAL MEDICAL CONVENTION.

In a notice of the National Medical Convention which met in New York in May last, our respected contemporary, *the Southern Journal of Medicine and Pharmacy*, has published the following as one of the resolutions *adopted* by that body, viz.:

"*Resolved*, That the union of the business of *Teaching* and *Licensing* in the same hands, is wrong in principle, and liable to great abuse in practice. Instead of conferring the right to license on Medical Colleges, and State and County Medical Societies, it should be restricted to one Board in each State, composed in fair proportion of representatives from its Medical Colleges and the profession at large, and the pay for whose services as Examiners should in no degree depend on the number licensed by them."

This is an error. According to the official report of the proceedings, which has been published in most of the medical journals, this *resolution* was proposed by Dr. Bartles, and after some discussion and several motions to amend, lay on the table, &c., was finally, on the motion of Dr. Manley, referred to a special committee. This committee, as appointed by the President of the Convention, consists of the following gentlemen, viz.: "Drs. McNaughton, Albany, N. Y.; J. R. Manley, N. Y.; J. W. Francis, N. Y.; Isaac Parrish, Philadelphia; R. Blakeman, Fairfield, Conn.; J. Cullen, Richmond, Va.; and Thomas Cock, N. Y." The Committee, it is understood, will report to the Convention to be held in Philadelphia in May, 1847.

### HEALTH OF PHILADELPHIA.

There is scarcely a spot on the globe, probably, where the inhabitants more uniformly enjoy the blessing of good health, and where consequently the Bills of Mortality are lower, than in the City of Philadelphia; on this account we have not been in the practice of noticing the subject, except on one or two occasions during the late epidemic of small pox. The unusual number of interments reported by the Board of Health which occurred during the week extending from the 11th to the 18th inst., having occasioned considerable remark, we are induced to publish the *report*, that our readers may see the ages of the deceased, and the diseases of which they died. From this it will be seen that there were 251 interments, of which 157 were children under ten years of age. It will also be seen that of these 46 died of cholera infantum; 18 of convulsions; 7 of marasmus; and 14 were still-born. So that an inspection of the report will show that there is no particular epidemic prevailing, except that of the summer complaint of children. The number of *fever* cases is unusually small, and with the exception we have mentioned, the inhabitants of the city, we believe, never enjoyed better health than at the present time.

Moreover, it must be recollected that this is the season of greatest mortality in our American cities generally. It occurs chiefly among children, and in large proportion among those of the poorer classes. The abundance of unripe fruit and green vegetable food brought to market, and the elevated temperature during the summer months, may be regarded as the great predisposing and exciting causes.

During the corresponding week, the interments in the City of New York were 425; and in St. Louis and other western as well as eastern cities, the mortality has been quite in the proportion of that of Philadelphia and New York. Two or three weeks, however will bring cooler nights, and consequently relief to the little sufferers.

### HEALTH OFFICE.

PHILADELPHIA, July 18, 1846.

Interments in the City and Liberties of Philadelphia, from the 11th to the 18th July.

| DISEASES. | Adults. | Children. |
|---|---|---|
| Apoplexy, | 6 | 0 |
| Burns, | 0 | 1 |
| Cancer, | 1 | 0 |
| —— of the Stomach, | 1 | 0 |
| Casualties, | 1 | 1 |
| Croup, | 0 | 1 |
| Congestion of the Lungs, | 2 | 0 |
| —— of the Brain, | 4 | 0 |
| Cholera Infantum, | 0 | 46 |
| —— Morbus, | 2 | 2 |
| Consumption of the Lungs, | 10 | 2 |
| Convulsions, | 1 | 18 |
| —— Puerperal, | 1 | 0 |
| Cyanosis, | 0 | 1 |
| Diarrhœa, | 3 | 4 |
| Dropsy, | 2 | 0 |
| —— Heart, | 0 | 1 |
| —— of the Head, | 0 | 5 |
| —— in the Breast, | 1 | 1 |
| Disease of the Brain, | 1 | 1 |
| —— of the Heart, | 1 | 0 |
| —— of the Lungs, | 1 | 0 |
| —— of the Bowels, | 0 | 1 |
| Drowned, | 1 | 4 |
| Dysentery, | 4 | 2 |
| Debility, | 0 | 4 |
| Effusion on the Brain, | 1 | 1 |
| —— on the Lungs, | 0 | 1 |
| Epilepsy, | 1 | 0 |
| Erysipelas, | 0 | 1 |
| Excessive Heat, | 6 | 1 |
| Fever, Bilious, | 1 | 0 |
| —— Cerebral, | 1 | 0 |
| —— Intermittent, | 0 | 1 |
| —— Nervous, | 2 | 0 |
| —— Puerperal, | 2 | 0 |
| —— Scarlet, | 0 | 5 |
| —— Typhus, | 1 | 0 |
| —— Typhoid, | 1 | 0 |
| Hæmorrhage from Bowels, | 0 | 1 |
| —— from Uterus, | 1 | 0 |
| —— from Lungs, | 1 | 0 |
| Ileus, | 1 | 0 |
| Inflammation of the Brain, | 1 | 6 |
| —— of the Breast, | 0 | 1 |
| —— of the Bronchi, | 0 | 2 |
| | — | — |
| Carried over, | 63 | 115 |

| DISEASES. | Adults. | Children. |
|---|---|---|
| Brought over, | 63 | 115 |
| Inflammation of the Lungs | 2 | 3 |
| —— of the Stomach & Bowels, | 2 | 5 |
| —— of the Bladder, | 1 | 0 |
| —— of the Liver, | 1 | 0 |
| —— of Peritonæum, | 0 | 1 |
| Intussusception, | 0 | 1 |
| Inanition, | 0 | 2 |
| Mania a Potu, | 2 | 0 |
| Malformation of the Liver, | 0 | 1 |
| Marasmus, | 0 | 7 |
| Neuralgia, | 1 | 0 |
| Old Age, | 3 | 0 |
| Palsy. | 5 | 0 |
| Scirrhus, | 1 | 0 |
| Scrofula, | 1 | 1 |
| Sore Mouth, | 0 | 3 |
| Small Pox, | 0 | 1 |
| Still Born, | 0 | 14 |
| Strangulation, | 0 | 1 |
| Suicide, | 1 | 0 |
| Syphilis, | 1 | 0 |
| Tabes Mesenterica, | 0 | 1 |
| Teething, | 0 | 1 |
| Ulcerated Sore Throat, | 0 | 1 |
| Unknown, | 3 | 2 |
| Whooping Cough, | 0 | 4 |
| | — | — |
| | 87 | 164 |

| | |
|---|---|
| Under 1 year, | 108 |
| From 1 to 2 | 27 |
| 2 to 5 | 12 |
| 5 to 10 | 10 |
| 10 to 15 | 2 |
| 15 to 20 | 5 |
| 20 to 30 | 29 |
| 30 to 40 | 11 |
| 40 to 50 | 9 |
| 50 to 60 | 10 |
| 60 to 70 | 15 |
| 70 to 80 | 8 |
| 80 to 90 | 3 |
| 90 to 100 | 1 |
| 100 to 110 | 1 |
| | — |
| Total, | 251 |

Of the above there were 7 from the Almshouse, and21 people of colour, which are included in the total amount.

By order of the Board of Health.

SAMUEL P. MARKS, Clerk.

STATE OF THE THERMOMETER.

| | 9 o'clock. | 12 o'clock. | 3 o'clock. |
|---|---|---|---|
| July 12 | 85 | 94 | 98 |
| 13 | 79 | 84 | 88 |
| 14 | 78 | 83 | 85 |
| 15 | 67 | 71 | 72 |
| 16 | 65 | 72 | 73 |
| 17 | 66 | 65 | 63 |
| 18 | 64 | 69 | 73 |

BRAITHWAITE'S RETROSPECT.

The last number of *Braithwaite's Retrospect* (January to June) contains *a Synopsis*, or short abstract of the practical points and directions embraced in the articles it contains. This is a new feature in the work, which is itself a synopsis of the matters contained in the various medical journals, of which we now have a further synopsis—an abstract of abstracts!

There is no end to the labour saving of the present day. Mental, like physical aliment, is not only collected and prepared for the use of the needy, but it is ready chewed, and even digested, so that the indolent have nothing to do but to swallow!

In the number of the London Medical Gazette for June 5, 1846, there is the translation of an article on *Kiestein* or *Kyestein*, or, as the writer has it, *Kystein*, by Dr. Möller, of Königsberg, in which the author alludes to the researches of our friend and correspondent, Dr. E. K. Kane, but assigns the credit of them to Dr. Kane of Dublin! No correction is given either by the Translator or Editor of the Gazette. The results of M. Möller's researches correspond greatly with those of Dr. Kane. In a subsequent number—for June 12, 1846—is an account taken from the Gazette des Hôpitaux, of the "singular case" of "a physician killed by taking the medicine which he had prescribed for a patient." The sufferer is said to have been "a Dr. Bader, an old and respectable practitioner of Macou." We doubt not that the case will be copied into other journals, perhaps into some on this side of the Atlantic; who may not suspect—under their foreign aspect—that the real sufferer was a Dr. Baber, of Macon, Georgia!

